# Characterization of the *Pichia* *pastoris* Protein-*O*-mannosyltransferase Gene Family

**DOI:** 10.1371/journal.pone.0068325

**Published:** 2013-07-01

**Authors:** Juergen H. Nett, W. James Cook, Ming-Tang Chen, Robert C. Davidson, Piotr Bobrowicz, Warren Kett, Elena Brevnova, Thomas I. Potgieter, Mark T. Mellon, Bianka Prinz, Byung-Kwon Choi, Dongxing Zha, Irina Burnina, John T. Bukowski, Min Du, Stefan Wildt, Stephen R. Hamilton

**Affiliations:** Biologics Discovery (GlycoFi Inc.), Merck Research Laboratories, Lebanon, New Hampshire, United States of America; University of Exeter, United Kingdom

## Abstract

The methylotrophic yeast, *Pichia* *pastoris* , is an important organism used for the production of therapeutic proteins. However, the presence of fungal-like glycans, either N-linked or O-linked, can elicit an immune response or enable the expressed protein to bind to mannose receptors, thus reducing their efficacy. Previously we have reported the elimination of β-linked glycans in this organism. In the current report we have focused on reducing the O-linked mannose content of proteins produced in *P* *. pastoris* , thereby reducing the potential to bind to mannose receptors. The initial step in the synthesis of O-linked glycans in *P* *. pastoris* is the transfer of mannose from dolichol-phosphomannose to a target protein in the yeast secretory pathway by members of the protein-*O*-mannosyltransferase (*PMT*) family. In this report we identify and characterize the members of the *P* *. pastoris* *PMT* family. Like *Candida albicans*, *P* *. pastoris* has five *PMT* genes. Based on sequence homology, these PMTs can be grouped into three sub-families, with both *PMT1* and *PMT2* sub-families possessing two members each (*PMT1* and *PMT5*, and *PMT2* and *PMT6*, respectively). The remaining sub-family, *PMT4*, has only one member (*PMT4*). Through gene knockouts we show that *PMT1* and *PMT2* each play a significant role in O-glycosylation. Both, by gene knockouts and the use of Pmt inhibitors we were able to significantly reduce not only the degree of O-mannosylation, but also the chain-length of these glycans. Taken together, this reduction of O-glycosylation represents an important step forward in developing the *P* *. pastoris* platform as a suitable system for the production of therapeutic glycoproteins.

## Introduction

Recent advances in genomics and proteomics have fuelled the increasing demand for large quantities of therapeutic proteins. Because the majority of these therapeutically relevant proteins require certain posttranslational modifications, such as N-glycosylation or O-glycosylation for proper function [1], most glycoproteins of commercial importance are currently expressed in mammalian cell culture. Fungal protein-expression systems are viewed as a potential alternative because of high volumetric productivity [2,3], low media cost, lack of retroviral contamination and ease of generation of stable cell lines. However, the presence of high mannose-type N- and O-glycans renders glycoproteins derived from fungal expression systems less desirable for human applications [4]. Recently the methylotrophic yeast *Pichia* *pastoris* has become a model system for protein expression, and our lab has described the humanization of the *N*-glycosylation pathway of this organism [5]. However, currently little has been reported on the engineering of O-linked glycans in this organism.

**Figure 1:**
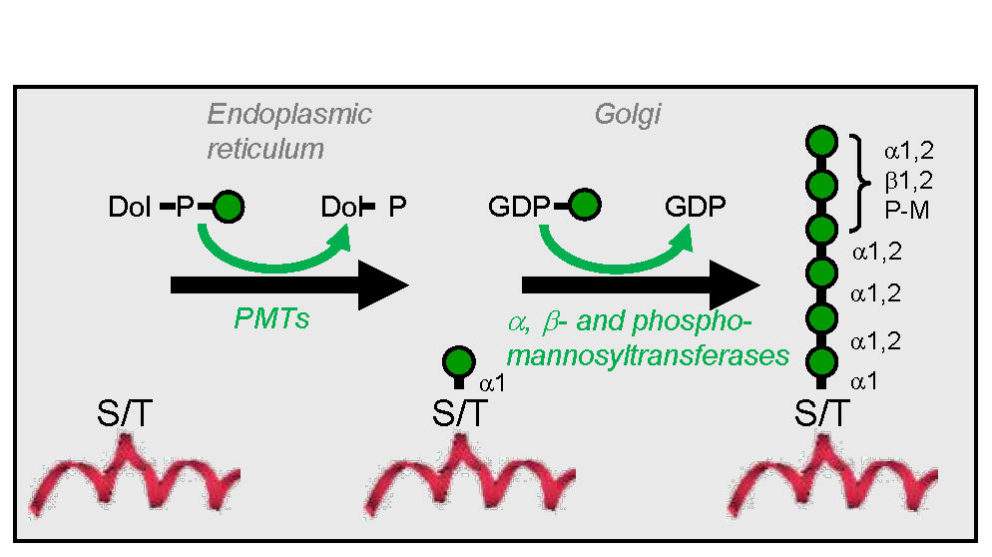
Illustrative representation of O-glycosylation in wild type *Pichia* *pastoris* . The diagram represents the O-glycosylation events that take place in the secretory pathway of *Pichia* *pastoris* . Following translocation of a protein into the endoplasmic reticulum, a mannose residue can be transferred onto a serine or threonine residue from Dol-P-Man by a member of the protein-*O*-mannosyltransferase (PMT) family. Subsequently, in the Golgi, this single mannose residue can be extended by the addition of one or more mannose residues by a combination of α-, β- or phospho-mannosyltransferases. Mannose residues are represented by *green circles*.

Protein O-mannosylation in fungi is initiated at the endoplasmic reticulum (ER) by a family of protein-*O*-mannosyltransferases (PMTs) that transfer mannose from dolichyl phosphate-activated mannose (Dol-P-Man) to serine or threonine residues of proteins entering the ER. In *P* *. pastoris* , additional mannosyltransferases then extend the mannose chain by up to four more residues [6] with GDP mannose serving as the carbohydrate donor (see Figure 1). The yeast *Saccharomyces cerevisiae*, where PMTs have been studied extensively for years, contains a highly redundant *PMT* gene family with up to seven members. They can be phylogenetically grouped into the three subgroups, *PMT1* (containing *PMT1*, *PMT5* and *PMT7*), *PMT2* (containing *PMT2*, *PMT3* and *PMT6*) and *PMT4* (as the sole member of this group), encoding proteins with different protein substrate specificities [7]. In *S. cerevisiae*, the major transferase activity is performed by heterodimeric Pmt1p and Pmt2p subfamily member complexes and Pmt4p homodimeric complexes [7]. Orchard and colleagues described rhodanine-3-acetic acid (RAA) derivatives as potent and specific inhibitors of *Candida albicans* Pmt1p [8,9]. In *S. cerevisiae*, however, it was shown that these inhibitors affect the members of all three *PMT* subfamilies [10]. Here we describe the five members of the *PMT* family of *P* *. pastoris* . We provide data on the phenotypes of individual gene knockouts and the effect previously described inhibitors have on them. We also show that these inhibitors not only reduce O-glycan site occupancy but also O-glycan chain length via an unknown mechanism.

## Materials and Methods

### Strains and DNA manipulations

*Escherichia coli* strain DH5α was used for recombinant DNA work. *P* *. pastoris* strains NRRL-Y11430 (wild type), PBP33 (a *Δoch1* derivative of NRRL-Y11430) or YGLY11 (a *Δpno1*, *Δbmt2*, *Δmnn4L1* derivative of NRRL-Y11430) were used for construction of yeast strains. PCR reactions were performed according to suppliers’ recommendations, using ExTaq™ (Takara Bio, Mountain View, CA), Taq Poly (Promega, Madison, WI) or *Pfu* Turbo^®^ (Stratagene, La Jolla, CA). Restriction and modification enzymes were from New England Biolabs (Ipswich, MA). PCR analysis of the *PMT* knockout strains was performed as described in [11]. The program used to determine the *P* *. pastoris* *PMT* homologues of the corresponding *S. cerevisiae* genes was BLAST [12]. The partial genomic sequence of *P* *. pastoris* was provided by Integrated Genomics Inc., with a public version of the *Pichia* genome now available at http://www.Pichiagenome.org/.

### Transformation of yeast strains

Yeast transformations were performed as described in [11]. In short, yeast cultures in logarithmic growth phase were washed twice in distilled water and once in 1 M sorbitol. 5–50 µg linearized DNA in 10 µl (TE: 10 mM Tris, 1 mM EDTA, pH 8.0) were mixed with 100 µl yeast cells and electroporated, using α BTX electroporation system (BTX, San Diego, CA). After addition of 1 ml recovery medium (1% yeast extract, 2% peptone, 2% dextrose, 4 ×10^−5^% biotin, 1 M sorbitol, 0.4 mg/ml ampicillin, 0.136 mg/ml chloramphenicol), the cells were incubated without agitation for 4 h at room temperature and then spread onto appropriate media plates.

### Construction of expression plasmids

The plasmid expressing the human plasminogen domains Kringle 1-3 was constructed the following way: Primers K1-3/UP (5'-CGGAATTCTCAGAGTGCAAGACTGGGAATAGAA-3') and K1-3/LP1 (5'-ATGATGATGACCACCACCGTCCTGGACCACAGGGGTTAG-3') were used to amplify the K1-3 domain from a plasmid encoding human plasminogen (a gift from Nick Menhart, Illinois Institute of Technology, Chicago) and add an *Eco*RI site at the 5’-end and a GGGHHH tag at the 3’-end (marked by italics). The resulting PCR product was then re-amplified using K1-3/UP and primer K1-3/LP2 (5'-TTAATGATGATGATGATGATGATGATGATGACCACCACC-3'), extending the His-tag to 9 units and adding a stop codon. The resulting product was then cloned into plasmid pCR2.1 TOPO (Invitrogen (now Life Technologies), Carlsbad, CA) to yield pBK105. In order to remove the N-glycosylation site in the K3 domain (position 308), the asparagine at this position was mutated to serine by inverse PCR, using pBK105 as a template and primers K3f (5'-ACCCCTCACACACATT C TAGGACACCAGAAAACTTC-3', the serine codon is underlined) and K3r (5'-CTG TGCACTCCAGTGCTGACAGGTGTG-3'). The resulting PCR product was then ligated to produce plasmid pBK118. Subsequently, the K1-3 insert was sequenced to confirm the mutation. Plasmid pBK118 was then digested with *Eco*RI, and the insert was cloned into the *Eco*RI site of pPICZalphaA (Invitrogen (now Life Technologies), Carlsbad, CA) to yield pBK119 (the K1-3 expression plasmid). Before transformation into yeast strains, pBK119 was linearized with *Pme*I. This facilitates integration of the vector into the *AOX1* promoter locus. Two plasmids expressing an IgG1 were constructed the following way: Construction of pDX580, which directs integration of the antibody genes into the *AOX2* locus of *P* *. pastoris* : The constant regions of the heavy chain (human IgG1) as well as the light chain (human Kappa) were codon optimized for expression in *P* *. pastoris* and synthesized by Life Technologies/ GeneArt AG (Regensburg, Germany). Variable regions were made in-house by overlap PCR using oligonucleotides purchased from IDT Inc. (Coralville, IA). Full length heavy and light chains with respective signal sequences (*Aspergillus niger* α-amylase for heavy chain, and chicken lysozyme for light chain) and restriction sites (*Bst*BI on the 5’-end and *Not*I on the 3’-end) were assembled by overlap PCR, and the resulting heavy and light chains were cloned into pCR2.1 TOPO to generate pDX344 and pDX349 respectively. pDX344 was then digested with *Bst*BI and *Not*I and the IgG1 heavy chain was cloned into pPICZA creating pDX518. Similarly, the light chain was released by *Bst*BI and *Not*I digest and also cloned into pPICZA, creating pDX490. To allow for integration into the *P* *. pastoris* genome, a plasmid was created by cloning a 1.5 kb fragment of the *PpAOX2* promoter into the *Bgl*II / *Eco*RI sites of pPICZA, effectively replacing the *AOX1* promoter region with that of *AOX2*. This plasmid was named pBK85. The heavy chain cassette, encompassing the *AOX1* promoter, the antibody heavy chain and the *AOX1* terminator, was then released from pDX518 by *Bgl*II / *Bam*HI digest and cloned into the *Bam*HI site of pBK85 creating pDX564. The light chain cassette, encompassing the *AOX1* promoter, the antibody light chain and the *AOX1* terminator, was then released from pDX490 by *Bgl*II / *Bam*HI digest and cloned into the *Bam*HI site of pDX564 creating the final IgG1 expression plasmid pDX580. This plasmid can be integrated into the *AOX2* promoter genomic region by linearization at the unique *Spe*I site in the plasmid and transformation into *P* *. pastoris* . Construction of pGLY2988, which directs integration of the antibody genes into the *TRP2* locus of *P* *. pastoris* : Expression/integration plasmid vector pGLY2988 contains expression cassettes under control of the methanol-inducible *P* *. pastoris* *AOX1* promoter that encode the heavy (Hc) and light (Lc) chains of an IgG1. The IgG1 Hc and Lc fused at the N-terminus to the *S. cerevisiae* α-MAT pre signal peptide were synthesized by Life Technologies/ GeneArt AG (Regensburg, Germany). Each was synthesized with unique 5' *Eco*RI and 3' *Fse*I sites. Both nucleic acid fragments encoding the Hc and Lc proteins fused to the α-MAT pre signal peptide were separately subcloned using 5' *Eco*RI and 3' *Fse*I unique sites into an expression plasmid vector pGLY2198, which contains the *P* *. pastoris* *TRP2* gene as targeting region and the Zeocin-resistance marker and generates expression cassettes under the control of the *AOX1* promoter and *S. cerevisiae CYC1* terminator, to form plasmid vectors pGLY2987 and pGLY2338, respectively. The Lc expression cassette was then removed from plasmid vector pGLY2338 by digesting with *Bam*HI and *Not*I, and subcloned into plasmid vector pGLY2987 digested with *Bam*HI and *Not*I, thus generating the final expression plasmid vector pGLY2988.

### Construction of PMT knockout strains

The *PpPMT* deletion alleles were generated either using the URA5-blaster system described in [13], or by the PCR overlap method [14] using primers designed based on the genomic sequence. In the first PCR reaction, DNA comprising about 1kb of nucleotide sequences for 5’- and 3’-regions flanking the *PMT* ORFs and the NAT, HYG or KAN resistance markers [15,16] were individually PCR amplified. *P* *. pastoris* genomic DNA (strain NRRL-Y11430) was used as a template for the *PpPMT* flanking regions, whereas NAT, HYG and KAN resistance marker fragments were amplified using plasmids as described in [15,16]. Then, in a second PCR reaction, the three first round PCR products per knockout were used as templates to generate an overlap product that contained all three fragments in a single linear allele. The final PCR product was then directly employed for transformation. Transformants were selected on YPD medium containing 200 µg/ml of hygromycin, 100 µg/ml of Nourseothricin, or 100 µg/ml of kanamycin. In each case the proper integration of the mutant allele was confirmed by PCR.

### Synthesis of Pmt inhibitors

Preparation of PMTi-1, (5-[[3,4-bis(phenylmethoxy) phenyl] methylene]-4-oxo-2-thioxo-3-thiazolidineacetic acid), was as follows: The procedure was adapted from Orchard et al. in U.S. Pat. No. 7,105,554 and the compound is referred to in [8] as **1c**. A solution of rhodanine-3-acetic acid (1 g, 5.20 mmol, 1 eq), 3,4-dibenzyloxybenzaldehyde (2.04 g, 6.25 mmol, 1.2 eq), and sodium acetate (1.3 g, 15.6 mmol, 3 eq) in acetic acid (30 mL) was heated to reflux, and stirred overnight. As the reaction mixture was cooled to room temperature, the product was precipitated and filtered and washed with acetic acid, then petroleum ether. The residue was dissolved in hot DMSO, filtered, and precipitated by addition of water. Upon cooling, the precipitate was filtered and recrystallized from ethyl acetate and petroleum ether to give a product which was suspended in water and freeze-dried overnight in vacuo to give the final product as a fluffy yellow powder.

Preparation of PMTi-3, (5-[[3-(1-Phenyl-2-hydroxy)ethoxy)-4-(2-phenylethoxy)] phenyl] methylene [-4-oxo-2-thioxo-3-thiazolidineacetic Acid), (Orchard et al. in U.S. Pat. No. 7,105,554) was synthesized in three steps as follows: Step 1: Production of (+)-(S)-2-Acetoxy-1-bromo-1-phenylethane. Cold HBr-acetic acid (12.4 g, 52.2 mmol) was added drop-wise to (-)-(R)-1-phenylethane-1,2-diol (2.4 g, 17.4 mmol) during about five minutes and the mixture was stirred at room temperature for 40 minutes. Water (25 mL) was added and the solution was neutralized with sodium carbonate and extracted with ether (3x30 mL). The combined extracts were dried and evaporated to give (+)-(S)-2-acetoxy-1-bromo-1-phenylethane (3.93 g, 93%), d25 1.415 g/mL. Step2: Production of 3-[(1-Phenyl-2-hydroxy) ethoxy]-4-(2-phenylethoxy)-benzaldehyde (2-Acetoxy-1 bromoethyl). benzene (3.32 g, 13.67 mmol, 1.2 eq) (the product of Step 1), was added to a stirred solution of 3-hydroxy-4-(2-phenylethoxy)-benzaldehyde (2.76 g, 11.39 mmol, 1 eq) and cesium carbonate (2.97 g, 9.11 mmol, 0.8 eq) in N,N-dimethylformamide (15 mL). The solution was stirred for 19 hours at room temperature, and then for 21 hours at 80°C. The reaction was worked up by partitioning between ethyl acetate and water (brine was added to help break up the emulsion that formed). The organic layer was washed twice more with water, brine, and then dried over sodium sulfate and evaporated to give a dark oil. The residue was purified by chromatography on silica gel, and elution with diethyl ether yielded an orange oil. This oil was dissolved in methanol (100 ml) and to the solution was added an aqueous solution of sodium hydroxide (7 mL, 1M). After 30 minutes, the mixture was evaporated (to remove the methanol) and the residue partitioned between dichloromethane and water. The organic layer was dried over sodium sulfate and evaporated. The residue was purified by chromatographed on silica gel, and elution with petroleum ether: diethyl ether (1:2) yielded the product as a cream colored powder. Step 3: Production of *PMT*i-3. A solution of rhodanine-3-acetic acid (158 mg, 0.828 mmol, 1 eq), 3-(1-phenyl-2-hydroxy)ethoxy)-4-(2-phenylethoxy) benzaldehyde (300 mg, 0.828 mmol, 1 eq) (the product of Step 2), and ammonium acetate (191 mg, 3 eq) in toluene (10 mL) was heated to reflux for 3.5 hours, cooled to room temperature, and diluted with ethyl acetate (50 mL). The organic solution was washed with 1M HCl (2x200 mL) and brine (200 mL) then dried over sodium sulfate and evaporated. After work-up, the residue was purified by chromatography on silica gel. Elution with ethyl acetate yielded a yellow gum, which was recrystallized from diethyl ether and petroleum ether to give the product as a yellow powder.

### Expression of reporter proteins

Protein expression for the transformed yeast strains was carried out either in shake flasks or 96 well deep well plates at 24°C with buffered glycerol-complex medium (BMGY) consisting of 1% yeast extract, 2% peptone, 100 mM potassium phosphate buffer pH 6.0, 1.34% yeast nitrogen base, 4x10^-5^% biotin, and 1% glycerol. The induction medium for protein expression was buffered methanol-complex medium (BMMY) consisting of 1% methanol instead of glycerol in BMGY. Pmt inhibitor PMTi-1 or PMTi-3 in methanol was added to the induction medium to the appropriate concentrations. Cells were harvested and centrifuged at 2000 rpm in a tabletop centrifuge for five minutes. For Western analysis, seven µl of the supernatant from the expression cultures was separated by polyacrylamide gel electrophoresis (SDS-PAGE) according to Laemmli [17] and then electroblotted onto nitrocellulose membranes (Schleicher & Schuell (now Whatman, Inc., Florham Park, N.J.)). Kringle 1-3 protein was detected on the Western blots using an anti-His antibody (H-15 at 1:300 dilution) from Santa Cruz Biotechnology Inc. (Santa Cruz, CA) and developed using the ImmunoPure Metal Enhanced DAB Substrate Kit (Pierce Biotechnology, Rockford, Ill.). The IgG1 was detected using an HRP-conjugated anti-human IgG (H&L at 1:1000 dilution) to detect heavy and light chains. O-glycan site occupancy and length was determined by high-performance anion exchange chromatography with pulsed amperometric detection (HPAEC-PAD).

### High-performance anion exchange chromatography with pulsed amperometric detection (HPAEC-PAD)

To measure O-glycan content, the protein of interest was purified from the growth medium using either nickel chelation chromatography (for K1-3) [18], or protein A (for the IgG1). Subsequently the O-glycans were released from the protein of interest by alkaline elimination (beta-elimination). This process also reduces the newly formed reducing terminus of the released O-glycan (either oligomannose or mannose) to mannitol. The mannitol group thus serves as a unique indicator of each O-glycan. 0.5 nmol or more of protein, contained within a volume of 100 µl PBS buffer, was required for beta elimination. The sample was treated with 25 µl alkaline borohydride reagent and incubated at 50°C for 16 hours. About 20 µl arabitol internal standard was added, followed by 10 µl glacial acetic acid. The sample was then centrifuged through a Millipore filter containing both SEPABEADS and AG 50W-X8 resin and washed with water. The samples, including wash, were transferred to plastic autosampler vials and evaporated to dryness in a centrifugal evaporator. 150 µl 1% AcOH/MeOH was added to the samples and the samples were evaporated to dryness in a centrifugal evaporator. This last step was repeated five more times. 200 µl of water was added and 100 µl of the sample was analyzed by high pH anion-exchange chromatography coupled with pulsed electrochemical detection-Dionex HPLC (HPAEC-PAD). Average O-glycan occupancy was determined based upon the amount of mannitol recovered. For a more detailed protocol please refer to [19].

### Microarray analysis

The genome of *Pichia* *pastoris* strain NRRL-Y11430 was sequenced by Agencourt (now Beckman-Coulter) and annotated by Biomax and will be reported in greater detail in an accompanying manuscript [Mickus et al, in preparation]. The annotated genome was used to generate Agilent microarray probes for all 5424 genes for 3' biased hybridization protocol to a density of 2-3 probes per gene (4207 genes with 3 probes/transcript and 1217 genes with 2 probes/transcript). This custom-designed Agilent *P* *. pastoris* 15 k 3.0 array (8x15K) gene microarray was used for all whole genome gene-chip RNA expression analyses. To generate the transcript analysis discussed herein of the PMT genes, the wild type strain NRRL-Y11430 was cultivated in quadruplicate in 0.5 L fermenters (Sixfors multifermentation system, ATR Biotech, Laurel, MD) with a standard glycerol-to-methanol batch/fed-batch process as described previously [20] with pH 6.5, 24°C, 300 ml airflow/min, and initial stirrer speed of 550 rpm, which increased to 1200 rpm linearly between hours 1 to 10 using IRIS multifermenter software (ATR Biotech, Laurel, MD). The batch phase was initiated in BMGY medium (Invitrogen, a subsidiary of Life Technologies, Carlsbad, CA) with an initial charge of 4% glycerol and cultivated until glycerol was exhausted, followed by a fedbatch methanol induction phase where a methanol solution comprised of 100% methanol, 5 mg/L biotin and 12.5 ml PTM1 salts was fed at 0.6 g/h. Samples were taken in quadruplicate by removing enough cell broth to obtain ~1x10^6^ cells, and each aliquot was centrifuged for 30 seconds at 10,000 x g and flash frozen. The frozen samples were shipped to Cogenics (now Beckman-Coulter Genomics, Morrisville, NC) for RNA extraction and microarray hybridization. Raw intensity values were normalized using standard statistical methods [21]. Normalized intensity values from four independent samples were averaged to generate the expression values reported. 

## Results

### 1): The *Pichia* *pastoris* genome contains five protein mannosyltransferases of which PMT2 is most highly expressed

**Figure 2:**
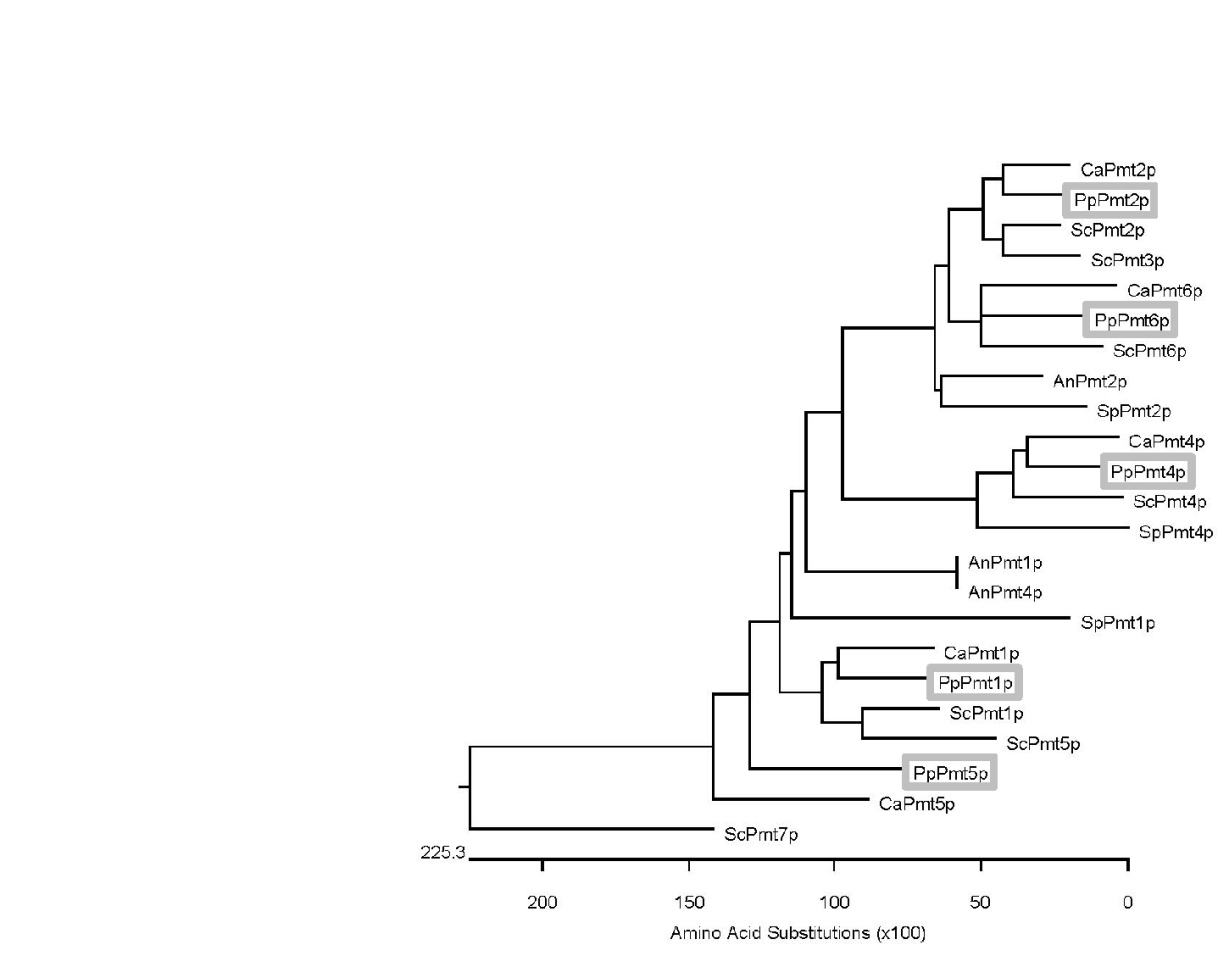
Phylogenetic analysis of *Pichia* *pastoris* Pmt proteins and related homologues. The phylogenetic tree was generated in the MegAlign application of the Lasergene Suite of applications (DNASTAR, Madison, WI) following CLUSTALW alignment. The scale at the bottom represents the evolutionary distance by amino acid substitutions. The PMT members from *Pichia* *pastoris* (PpPmt), *Candida albicans* (CaPmt), *Saccharomyces cerevisiae* (ScPmt), *Aspergillus nidulans* (AnPmt) and *Schizosaccharomyces pombe* (SpPmt) were compared. Sequences were derived from genomic databases (http://www.Pichiagenome.org/; http://www.yeastgenome.org/; http://www.broadinstitute.org/).

**Figure 3:**
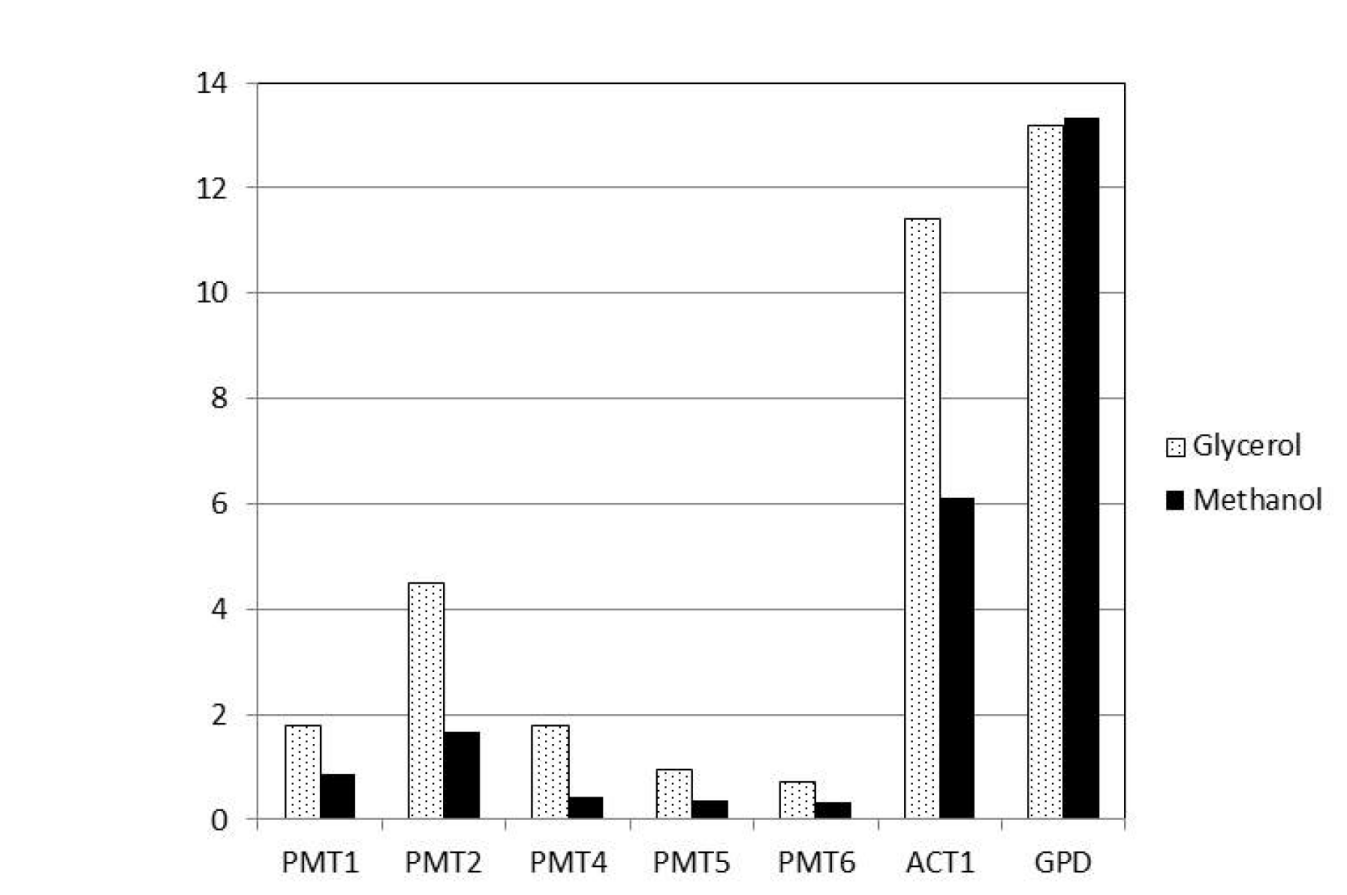
*PMT* expression profile by microarray analysis. The relative levels of PMT mRNA levels were assessed by microarray analysis under glycerol (*shaded bars*) or methanol (*black bars*) growth conditions as described in the Materials and Methods Section. mRNA levels for *ACT1* and *GPD* have been added for reference.

Querying a *P* *. pastoris* genomic sequence database with the open reading frames of all seven *S. cerevisiae* PMTs, we identified the *P* *. pastoris* *PMT1* (770 amino acids with 53% identity; Accession no. XP002491100), *PMT2* (750 amino acids with 64% identity; Accession no. XP002491148), *PMT4* (741 amino acids with 54% identity; Accession no. CCA36946), *PMT5* (741 amino acids with 35% identity; Accession no. XP002489942), and *PMT6* (752 amino acids with 51% identity; Accession no. XP002494221) homologues. No homologues for *ScPMT3* or *ScPMT7* were found. The importance of PMTs in fungal cell wall integrity have made them attractive targets for antifungal therapies, and their sequences have been elucidated in many biologically relevant species [10,22–25]. Phylogenetic analysis of the *P* *. pastoris* *PMT* genes compared with their homologues from *S. cerevisiae*, *Schizosaccharomyces pombe*, *Candida albicans* and *Aspergillus nidulans* showed that the *P* *. pastoris* *PMT* genes are most closely related to their *C. albicans* counterparts (see Figure 2). Similar to *P* *. pastoris* , the *PMT* gene family of *C. albicans* consists of five genes (*PMT1*, *PMT2*, *PMT4*, *PMT5* and *PMT6*) [22], whereas *A. nidulans* as well as *S. pombe* each only contain three *PMT* genes, one for each subgroup. To determine the expression level of the five identified *PMT* homologs in *P* *. pastoris* , an Agilent RNA expression microarray was employed. The wild type NRRL-Y11430 strain was cultivated in quadruplicate in 0.5 L fermentation vessels in a standard glycerol-to-methanol process that is typically used for heterologous protein production [20]. Samples were taken when the cultures reached 50 mg/ml of wet cell weight (approximately halfway through glycerol batch phase), at the end of batch phase, and then 4 h and 24 h into methanol induction. These samples were then applied to an Agilent array containing probes directed against the NRRL-Y11430 annotated geneset as described in an accompanying manuscript (Mickus et al., in preparation). From these whole genome expression data, normalized intensity values for the five annotated *PMT* genes was compared to the Actin (*ACT1*) and Glyceraldehyde-3-Phosphate Dehydrogenase (*GPD*) control genes and data are plotted for the glycerol and 24 h methanol time points (see Figure 3). Comparative expression levels varied significantly among the PMTs; however, transcripts for all of the genes were detected under all conditions tested with even the most weakly expressed family member (*PMT6*) in the ~60^th^ percentile of transcripts detected under both conditions. *PMT2* was the most highly expressed gene among the *P* *. pastoris* PMTs with *PMT1* and *PMT4* showing intermediate expression and *PMT5* and *PMT6* the weakest. This is consistent with what has been reported in *S. cerevisiae* where the Pmt1p/Pmt2p heterodimer accounts for the majority of O-mannosyl transfer. This is also consistent with genetic manipulation where elimination of Pmt2p activity resulted in the greatest effects both on viability and in terms of reducing O-mannosyl transfer to proteins. Expression of control genes is consistent with previous reports [26]. Notably, all five of the PMTs were dramatically repressed on methanol as compared to glycerol (Figure 3), which could simply be a function of reduced growth rate on methanol due, in turn, to a lower energy availability from methanol compared to glycerol, combined with a limiting feed rate [27].

### 2): Δpmt1 and Δpmt2 strains secrete protein with significantly reduced O-glycan site occupancy

**Figure 4:**
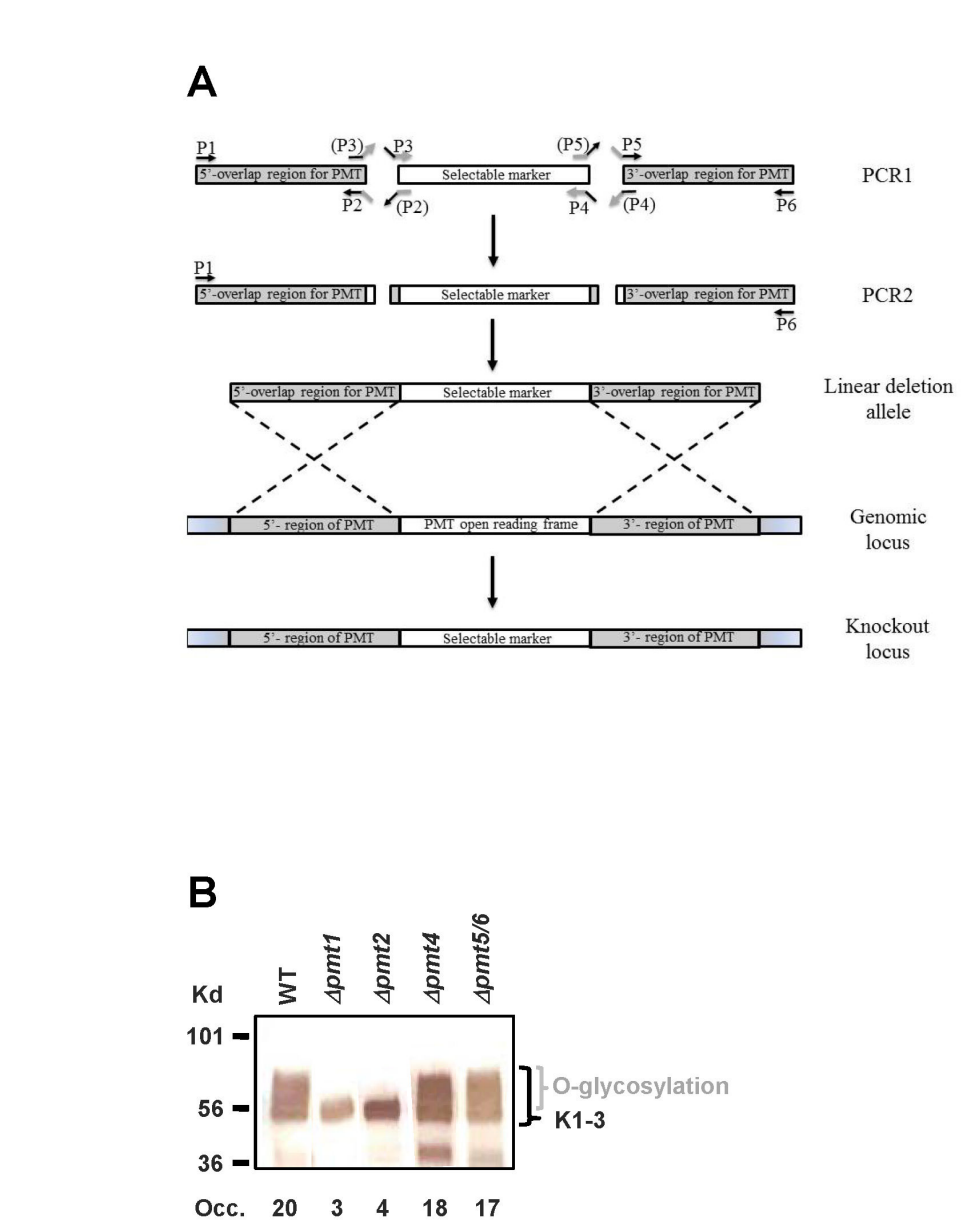
Overview of *PMT* knockout experiment. (A) The PCR overlap and *PMT* knockout procedure. First a linear gene deletion allele was constructed the following way: a region 5’ of the *PMT* ORF was amplified using primers 1 and 2, the selectable marker was amplified using primers 3 and 4, and a region 3’ of the *PMT* ORF was amplified using primers 5 and 6. The primers in parentheses were added to the schematic to illustrate that primers 2 and 3, as well as primers 4 and 5, are complementary to each other (thereby creating the overlaps). The amplified products were then used as templates together with primers 1 and 6 in the overlap reaction. The linear deletion allele was then integrated into the *P* *. pastoris* genome via homologous recombination, and knockouts were selected on plates supplemented with the appropriate antibiotic. (B) Western blot analysis of Kringle 1-3 protein (K1-3) expressed in wild type and *PMT* knockout strains. Strains JC53 (wild type), JC55 (*Δpmt1*), JC66 (*Δpmt2*), JC65 (*Δpmt4*), and JC51 (*Δpmt5/6*) were grown and induced in shake flasks. Seven µl of each supernatant was separated by polyacrylamide gel electrophoresis (SDS-PAGE) and then electroblotted onto nitrocellulose membranes. K1-3 protein was detected using an anti-His antibody. O-glycan site occupancy (Occ.) was determined as described in Materials and Methods. The expected mass of the fully deglycosylated protein is 44.2 kDa, and the band at 36 kDa probably represents proteolytic cleavage.

**Figure 5:**
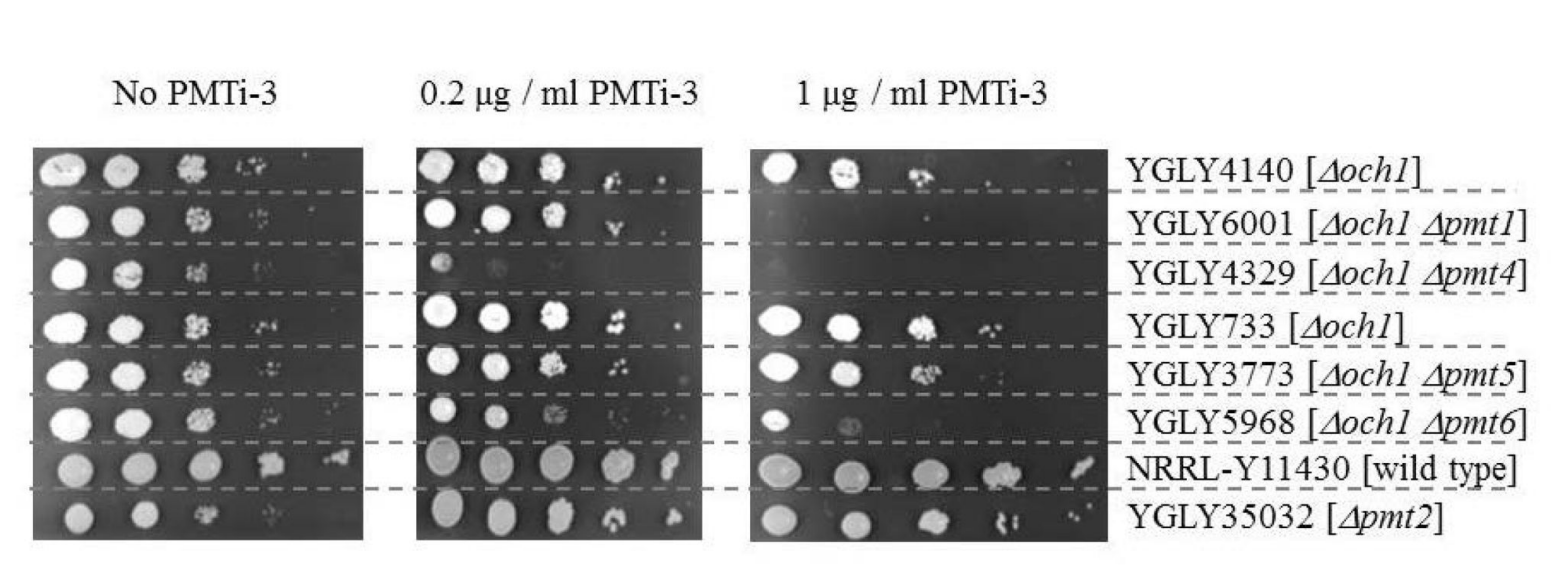
Inhibitor sensitivity of *PMT* knockouts. For dilution assays, yeast cells were grown overnight in YSD and then adjusted to an OD600 of 1.0 using YSD, diluted in 10-fold increments, and then 3 µl of each dilution were spotted onto solid YSD media with varying concentrations of PMTi-3. The picture of the plate without PMTi-3 was taken after 48 hours, and the picture of the plates containing PMTi-3 were taken after 96 hours of incubation at 25^°^C. YGLY4140 is the parent strain of YGLY6001 and YGLY4329, YGLY733 is the parent strain of YGLY3773 and YGLY5968, and NRRL -Y11430 is the parent strain of YGLY35032.

In order to determine which of the *PMT* genes in *P* *. pastoris* are responsible for the addition of O-glycans on secreted protein, we created strains with individual knockouts for *PMT1*, *PMT2*, and *PMT4*, and a strain with a double knockout of both *PMT5* and *PMT6* as described in Materials and Methods, and shown in the schematic in Figure 4A. It has been reported that in *C. albicans*, *S.* pombe and even *P* *. pastoris* an individual knockout of the *PMT2* gene is lethal [22,24]; however we were able to construct single knockout strains of all five *Pichia* *pastoris* *PMT* genes, but not double knock-outs *Δpmt1/Δpmt2*, *Δpmt1/Δpmt4* or *Δpmt2/pmt4*. Not unexpectedly, we also found that the *Δpmt1* and *Δpmt4* strains displayed a slight and the *Δpmt2* strain a significant slow growth phenotype (see Figure 5). In order to determine the feasibility of *PMT* knockouts in N-linked glycosylation engineered strains, we also attempted the *PMT* deletions in a *Δoch1* background with reduced N-glycan complexity. We were able to generate the *PMT1*, *PMT4*, *PMT5* and *PMT6* single knockouts as well as the *PMT5/PMT*6 double knockouts, but we were unable to obtain a *Δpmt2* strain (for a list of strains and their corresponding genotype please see Table 1). The *PMT1*, PMT2, and *PMT4* single knockouts, as well as the *PMT5/PMT6* double knockout strains were then transformed with a plasmid expressing a His-tagged reporter protein consisting of human plasminogen domains Kringle 1, Kringle 2 and Kringle 3 (K1-3) under the control of the *P* *. pastoris* alcohol oxidase 1 (*AOX1*) promoter. Since the Kringle 3 domain contains an N-glycosylation site at position 308, we changed this asparagine residue to a serine by site directed mutagenesis in order to make O-glycosylation the only post-translational modification of the secreted protein. The protein was then expressed in shake flasks and analyzed by SDS-gel analysis followed by Western blotting for size. Furthermore, we removed the bound O-glycans by beta elimination and analyzed them by high pH anion-exchange chromatography coupled with pulsed amperometric detection-DIONEX HPLC (HPAEC-PAD) for O-glycan site occupancy determination (see Materials and Methods). As shown in Figure 4B, only the *PMT1* and *PMT2* knockouts lead to a significant reduction in the extent of O-glycosylation, corresponding to a reduction in O-glycan site occupancy from 20 down to 3 to 4 moles of O-linked glycan per mole of protein (Table 2). By contrast, the *PMT4* single knockout and the *PMT5/PMT6* double knockout only displayed a very slight reduction of about 10% in O-glycan site occupancy (Table 2). We therefore conclude that in *P* *. pastoris* Pmt1p and Pmt2p are the main transferases responsible for O-glycan attachment to secreted protein.

**Table 1:** *P* *. pastoris* strains used in this study.

Strain	Relevant genotype
NRRL-Y11430	wild type
YGLY4280	*TRP2*:Zeo^R^/IgG1
YGLY4140	*Δoch1*::lacZ
YGLY6001	*Δoch1*::lacZ *Δpmt1*::Nat^R^
YGLY4329	*Δoch1*::lacZ *Δpmt4*::Nat^R^
YGLY733	*Δoch1*::lacZ
YGLY3773	*Δoch1*::lacZ *Δpmt5*::lacZ-URA5-LacZ
YGLY5968	*Δoch1*::lacZ *Δpmt6*::lacZ-URA5-lacZ
YGLY35032	*Δpmt2*::Hyg^R^
YJC53	*AOX1*:Zeo^R^/K1-3 *Δoch1*::lacZ-*URA3*-lacZ
YJC55	*Δpmt1*::Hyg^R^ *AOX1*:Zeo^R^/K1-3 *Δoch1*::lacZ-*URA3*-lacZ
YJC66	*Δpmt2*::Nat^R^ *AOX1*:Zeo^R^/K1-3
YJC65	*Δpmt4*::Nat^R^ *AOX1*:Zeo^R^/K1-3
YJC51	*Δpmt5*::Hyg^R^ *Δpmt6*::Kan^R^ *AOX1*:Zeo^R^/K1-3
YJC204	*AOX2*:Zeo^R^/IgG1
YJC200	*Δpmt1*::Hyg^R^ *AOX2*:Zeo^R^/IgG1
YJC210	*Δpmt2*::Nat^R^ *AOX2*:Zeo^R^/IgG1

**Table 2:** O-glycan site occupancy of *PMT* knockout strains (w or w/o PMTi).

Strain	Relevant genotype	PMTi	O-glycan site occupancy	
NRRL-Y11430	wild type	–	20	
		+	9	
YJC55	*Δpmt1*	–	3	
		+	2	
YJC66	*Δpmt2*	–	4	
		+	4	
YJC65	*Δpmt4*	–	18	
		+	7	
YJC51	*Δpmt5 Δpmt6*	–	17	
		+	6	

O-glycan site occupancy of K1-3 protein expressed in wild type and *PMT* knockout strains in the presence or absence of 2 µM PMTi-1. Occupancy is expressed as number of O-mannose chains per molecule of K1-3.

### 3): Pmt inhibitors block the activity of multiple PMTs in *Pichia* *pastoris*

In search for novel antifungal agents, Orchard and colleagues described in 2004 a group of rhodanine-3-acetic acid derivatives that inhibit Pmt1p activity in *C. albicans* [8]. The fact that these inhibitors are indeed specific for Pmt1p in *C. albicans* was later confirmed by Cantero and coworkers [9]. In *S. cerevisiae*, however, they have been shown to affect members of all three subclasses [10]. In order to test whether these compounds could be used instead of *PMT* knockout strains and thereby circumvent the accompanying slow growth phenotype, we tested the effect these inhibitors have in wild type *P* *. pastoris* and a subset of the *PMT* knockout strains described above. To this end, we grew strains yJC53 (wild type for *PMT*), yJC55 (*Δpmt1*), yJC66 (*Δpmt2*), yJC65 (*Δpmt4*) and yJC51 (*Δpmt5/Δpmt6*) in the absence or presence of 2 µM PMTi-1 (described in [8]) and analyzed the purified K1-3 protein for O-glycan site occupancy (see Table 2). Whereas PMTi-1 leads to a substantial reduction in O-glycan site occupancy in *PMT* wild type, and the *Δpmt4* single and *Δpmt5/Δpmt6* double knockout strains, it had no apparent effect on the *Δpmt1* and *Δpmt2* strains. In a related experiment we determined the growth characteristics of single *PMT* knockout strains on media containing varying amounts of a slightly modified compound, PMTi-3, with approximately ten times higher specific activity (for details please refer to Materials and Methods), using a drop dilution assay. As is evident from Figure 5, the *Δpmt4* strain is hypersensitive to PMTi-3, as it shows a significant growth defect already at low concentrations. At higher PMTi-3 concentrations the *Δpmt1* strain also is unable to grow and the *Δpmt6* strain displays a slow growth phenotype, whereas the growth of the *Δpmt2* and *Δpmt5* strains are unaffected. Taken together, this suggests that in *P* *. pastoris* , rhodanine-derived Pmt inhibitors affect multiple PMTs, and that different inhibitors are able to affect different PMTs (PMTi-1 inhibiting *PMT1* and *PMT2*, and PMTi-3 inhibiting *PMT2* and *PMT5*).

### 4): Pmt inhibitors block *O-*mannosylation at concentrations much below those at which growth is inhibited

**Figure 6:**
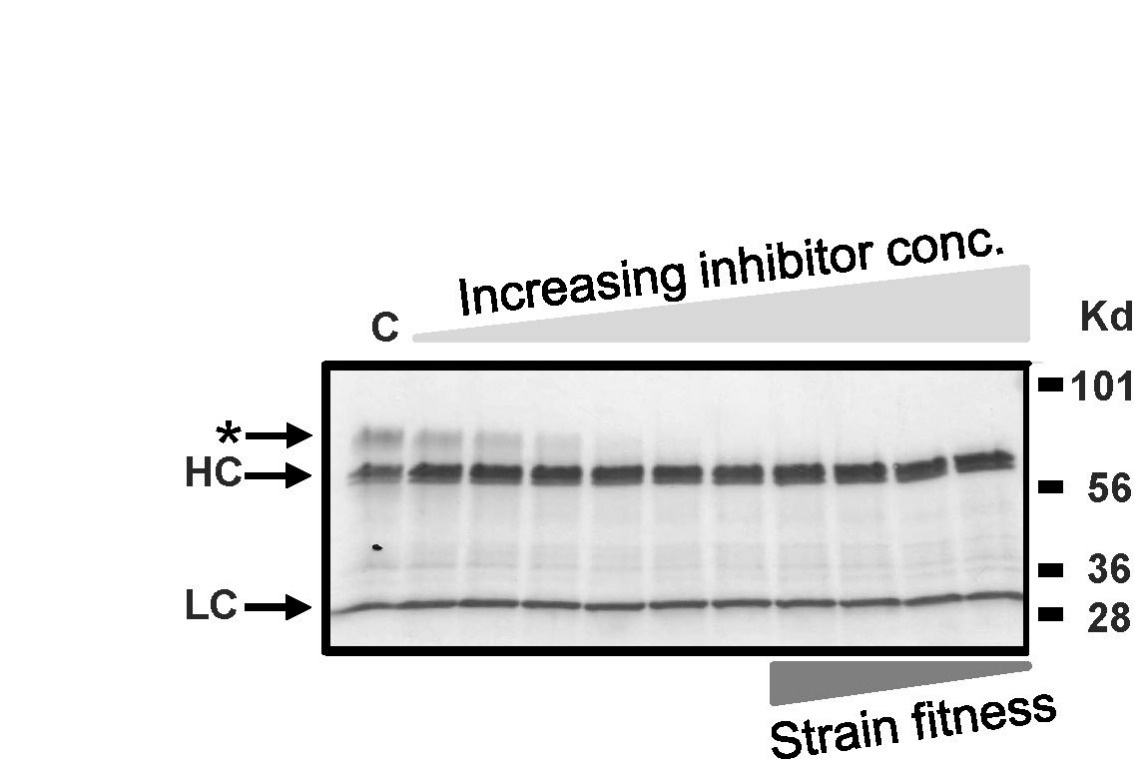
Western blot analysis of an IgG1 expressed in the presence of PMTi-3. The *PMT* wild type strain YGLY4280 was grown in 96 well deep-well plates and induced in medium containing no PMTi-3 (C) or increasing concentrations of PMTi-3 (0.0187, 0.0375, 0.075, 0.15, 0.3, 0.625, 1.25, 2.5, 5, 10 µg/ml). After overnight induction, 7 µl of each supernatant was separated by polyacrylamide gel electrophoresis (SDS-PAGE) and then electroblotted onto nitrocellulose membranes. IgG1 was detected using an anti-human IgG (heavy and light chain) antibody. Hyperglycosylated heavy-chain (indicated by an asterisk) is barely detectable at PMTi-3 concentrations of 0.15 µg/ml and higher, however strain growth and fitness (strain lysis) was still unaffected at PMTi-3 concentrations of up to 1.25 µg/ml. A representation of the PMTi-3 increase in concentration and the relative reduction in cell fitness are illustrated by *light* and *dark gray triangles* respectively.

For the industrial application of Pmt inhibitors in the production of therapeutic proteins it is of great importance that the inhibitors not only reduce O-mannosylation but also have a negligible effect on growth of the culture. Since relatively high concentrations of PMTi-1 are needed to consistently reduce O-glycan site occupancy in *P* *. pastoris* , we again employed PMTi-3 to test the window between reduction in O-glycan site occupancy and growth inhibition. To this end we grew strain YGLY4280 which expresses a recombinant IgG1(for genotype refer to Table 1) in non-inducing glycerol medium in 96 well plates overnight, and then induced the cultures in media containing PMTi-3 at increasing concentrations. After induction overnight, the supernatant was then analyzed by Western blot for the presence of O-glycosylated heavy and light chains. As can be seen in Figure 6, hyperglycosylated heavy chain (marked by an asterisk) is clearly visible in the control lane without inhibitor, but cannot be detected anymore in the sample incubated in the presence of 0.15 µg/ml of PMTi-3. Incidentally, PMTi-3 does not inhibit growth of *P* *. pastoris* at concentrations well above 1 µg/ml (see Figures 5 and 6). This means that there is about a 10 fold window in which PMTi-3 can be used to reliably reduce O-glycan site occupancy in *P* *. pastoris* .

### 5): PMT knockouts and Pmt inhibitors not only reduce O-glycan site occupancy but also O-glycan chain length in *P* *. pastoris*

For the production of therapeutic glycoproteins in the glycoengineered *P* *. pastoris* platform, it is essential to understand the O-glycosylation profile of secreted proteins fully. To this end, in addition to characterizing the O-glycan site occupancy following *PMT* manipulations, we also profiled the O-glycosylation structures under the same conditions. Table 3 shows that a *PMT2* knockout, and to a lesser extent a *PMT1* knockout, in addition to a reduction in O-glycan site occupancy also lead to shortened O-glycan chain lengths. When we compared these two parameters on protein that had been expressed in a wild type strain with PMTi-3 addition with those of protein expressed in *PMT1* and especially *PMT2* knockouts without PMTi-3 addition, we saw that this effect could also be accomplished by the addition of inhibitor during the induction phase (compare Table 3 line 2 with lines 3 and 5). Furthermore, the effect seems to be additive, as a *Δpmt2* strain induced in the presence of PMTi-3 expressed IgG1 with only single mannose O-glycan chains (Table 3 line 6).

**Table 3:** Occupancy and O-glycan length of *PMT* knockout strains (w or w/o PMTi).

Strain	*PMT* knockout	PMTi	Occupancy		Man1	Man2	Man3	Man4
YJC204	wild type	–	32		14	42	37	6
		+	6		76	24	0	0
YJC200	*Δpmt1*	–	14		34	26	33	6
		+	4		78	11	11	0
YJC210	*Δpmt2*	–	4		80	11	8	1
		+	<2		100	0	0	0

O-glycan site occupancy of an IgG1 expressed in wild type and *PMT* knockout strains in the presence or absence of 0.2 µM PMTi-3. Occupancy is expressed as number of O-mannose chains per molecule of antibody. Man1 through Man4 designates the percentage of mannose chains with the respective length.

## Discussion

In the current report we have identified and characterized the *PMT* family of *Pichia* *pastoris* . Comparing each member of the *P* *. pastoris* *PMT* family to those of several other yeast and fungal organisms, we have elucidated that the *P* *. pastoris* family bears close homology to that of *C. albicans* [10,22–25]. Both *C. albicans* and *P* *. pastoris* possess five *PMT* members. Like *C. albicans*, *P* *. pastoris* possesses homologues to *PMT1*, *2*, *4*, 5 and 6 but no homologues to *PMT3* or *PMT7* [22]. PMTs function as a dimeric complex. Typically a member of the *PMT1* sub-family (which includes *PMT1*, *PMT5* and *PMT7*) will dimerize with a member of the *PMT2* sub-family (which includes *PMT2*, *PMT3* and *PMT6*) to form a heterodimeric complex, or *PMT4* will self-dimerize to form a homodimeric complex [7]. As such, this implies that *P* *. pastoris* possesses a redundant member of both the *PMT1* and *PMT2* gene sub-families. To elucidate if each member of the *PMT1* and *PMT2* sub-families contributed equally to O-mannosylation, an Agilent RNA expression microarray was employed to determine the relative expression of all of the *PMT* genes. The normalized intensity values indicated that *PMT2* was predominantly expressed, while *PMT1* and *PMT4* were expressed to moderate extents and *PMT5* and *PMT6* expression were relatively the lowest. This was the case with strains utilizing either glycerol or methanol as a carbon source. Cantero and Ernst reported that *PMT2* and *PMT4* transcript levels were upregulated more than two fold in the presence of a Pmt1 inhibitor in *C. albicans* [28]. However we could not detect any significant change in expression level of any of the PMTs when grown in the presence of PMTi-3 (data not shown). The relative contribution of each *PMT* was further validated by generating knockouts of the five genes. Western blot analysis of protein produced in these knockouts indicated that both *PMT1* and *PMT2* were predominantly involved in O-linked mannose addition to the Kringle reporter protein, whereas *PMT4* knockout and the double knockout of both *PMT5* and *PMT6* showed only minor reduction in O-glycan site occupancy. Furthermore, *PMT2* was the only *PMT* that was demonstrated to be essential in a *Δoch1* N-glycan modified background. This fact was also one of the main reasons why a *PMT2* knockout was not a viable option to control O-glycosylation in glycoengineered strains. However, through the use of Pmt inhibitors it is possible to overcome this issue. The observation that *PMT2* is a highly essential gene has been previously reported for several yeast and fungi, including *C. albicans*, *S.* pombe, *A. fumigatus* and *Cryptococcus neoformans*, where *PMT2* knockout is lethal [22,24,29,30]. However, as in *S. cerevisiae* [25], no single *PMT* knockout is lethal in a wild type *P* *. pastoris* background.

During the revision of this manuscript, Govindappa and coworkers published a study on the role of the *PMT1* gene in O-mannosylation of insulin precursor in *P* *. pastoris* [31]. In agreement with our data they identified the same *P* *. pastoris* homologues of *S. cerevisiae PMT1*, *2*, *4*, *5* and *6*. In contrast to our results, however, they were unable to knock out the *PMT2* gene and therefore suggested it to be an essential gene. The reason for their inability to isolate a *PMT2* knockout despite screening approximately 1000 colonies is not obvious. The insertional inactivation method by which they tried to accomplish their *PMT2* knockout is based on a single crossover, or roll-in, integration into the genome [32]. This method leads to the duplication of part of the gene, which makes the integration potentially reversible. Our knock out method, however, is based on a double crossover integration, which leads to stable removal of the open reading frame of the gene to be knocked out [13]. It is also possible that the slow growth phenotype of the *PMT2* knockouts made the authors miss the much slower growing knocked out transformants, which in our case appeared several days after the wild type background colonies.

The data reported in our study indicates that Pmt1p and Pmt2p play the major role in modification of the Kringle reporter protein and that they most likely contribute to O-mannosylation of important cell house-keeping proteins. By contrast, *PMT4* demonstrated relatively little effect on Kringle modification but appeared to be essential for cell viability in either a *Δpmt1* or *Δpmt2* background. Therefore, it appears that while Pmt4p does not play a major role in O-mannosylation in a wild type strain background, it does play a vital role in the absence of Pmt1p or Pmt2p. This finding agrees with observations that have been made in other yeast and fungal organisms. In both *C. albicans* and *A. fumigatus*, the concerted knockout of *PMT1* and *PMT4* were lethal, like the single *PMT2* knockout in both of these organisms [22,29]. However, in *A. nidulans*, where *PMT2* was not essential, knockout of *PMT4* in combination with either *PMT*1 or *PMT2* proved lethal [23], as in *P* *. pastoris* . Likewise, both *PMT5* and *PMT6*, being paralogues of *PMT2* and *PMT1* respectively, may play more prominent roles in the absence of their sub-family members or under different stress conditions. Alternatively, both Pmt5p and Pmt6p may O-mannosylate specific proteins. For example, *S. cerevisiae* Pmt4p has been shown to O-glycosylate the protein gp115/Gas1p but not chitinase, thus indicating that protein O-glycosyltransferases differ in their specificity towards protein substrates [25].

Since many yeasts and fungi are pathogenic organisms, attempts have been made to produce anti-fungal agents to restrict their growth. In 2004, Orchard et al. identified a group of anti-fungal agents that were rhodanine-3-acetic acid (RAA) derivatives which targeted members of the *PMT* family [8]. In that study, the authors targeted *C. albicans* Pmt1p for anti-fungal development. A subsequent study in *C. albicans* concluded that the inhibitors preferentially blocked Pmt1p activity but could not exclude that the inhibitors had a minor effect on the other *PMT* family members [9]. By contrast, in *S. cerevisiae*, the RAA derivatives were shown to inhibit all three *PMT* sub-families, and not to be specific for Pmt1p [10]. In *P* *. pastoris* we demonstrated that RAA derivatives appear to target either Pmt1p and Pmt2p (in the case of PMTi-1) or Pmt2p and Pmt5p (in the case of PMTi-3) and have little effect on the other PMTs. As such, it was not unexpected to observe that *Δpmt*4 cells were hyper-sensitive to RAA derivatives, a similar phenotype to what was observed in *C. albicans* [9]. Furthermore, the hypersensitivity of this combination of inhibitor and knockout is consistent with the lethality of the double *Δpmt1*/*Δpmt4* mutants in *Candida* [22] and the fact that we were unable to generate either the *Δpmt1*/*Δpmt2*, the *Δpmt1*/*Δpmt4*, or the *Δpmt2*/*Δpmt4* double knockouts in *P* *. pastoris* .

Over the last decade significant advances have been made in engineering the N-linked glycosylation pathway of *P* *. pastoris* to produce glycoproteins possessing human-like N-glycans [5,18,33–35]. This process not only involved the generation of human-like N-glycans but also the elimination of non-desirable yeast glycans [36]. Likewise, it is desirable to limit yeast-type O-glycans on therapeutic proteins produced in this organism, and that the process utilized is scalable. Since the knockout of PMTs can lead to undesirable slow growth phenotypes, in the current study we have used a number of Pmt inhibitors which have demonstrated marked differences in their potencies. Although PMTi-1 was efficient at reducing O-glycan site occupancy, it also retarded growth at the dose required. A second inhibitor used, PMTi-3, proved much more potent and demonstrated significantly reduced O-mannosylation on a therapeutic antibody with little effect on cell growth. We used this therapeutic antibody as a second, commercially more relevant, test protein and to guard against the possibility that O-glycan reduction of K1-3 had been protein specific like has been shown for human insulin-like growth factor in *S. cerevisiae* [37]. RAA derivatives were also used to reduce the degree of O-mannosylation on a human anti-TRAIL-R antibody produced in the methylotrophic yeast *Ogataea* *minuta* [38]. Interestingly, these authors additionally observed an increase in the amount of assembled antibody secreted, and that antigen binding was also increased. The latter observation may have been attributable to the significant reduction in O-mannosylation observed on the light chain, which plays a key role in antigen binding. In contrast to this, a recombinant IgG1 produced in *P* *. pastoris* in the current report did not possess detectable O-mannosylation on the light chain, and incidentally no observable difference was detected in antigen binding when RAA derivatives were used (data not shown). In a parallel report by our laboratory a more potent RAA derivative (PMTi-4) was assessed for antibody production in *P* *. pastoris* [39]. Like the Kuroda et al. study, a dramatic increase in antibody titer was observed in the presence of RAA derivatives. Incidentally, unlike PMTi-3 which appears to target Pmt2p and Pmt5p, PMTi-4 preferentially inhibited Pmt2p [39].

Previous studies which have investigated *PMT* knockouts or the use of Pmt inhibitors have assessed cell fitness or reporter protein gel shifts to characterize these manipulations. Since our laboratory is concerned with the engineering of an expression platform for therapeutic protein production, we also characterized the glycan profiles resulting from our manipulations. An unexpected observation in our study was that in addition to reducing O-glycan site occupancy, both the knockout of *PMT1* or *PMT2*, or the use of PMTi-3, were shown to reduce O-glycan chain length. To our knowledge, this is the first characterization of a connection between *PMT* manipulations and O-linked mannose chain-length. Although we did not follow-up on the specific cause of chain-length reduction, there are a number of reasons why this might occur. For instance, the extension of the O-linked mannose chain is initially performed by members of the *KTR* α-1,2-mannosyltransferase gene family, which includes *KTR1*, *KTR3* and *KRE2* (reviewed for the *S. cerevisiae* homologues in [40]). It is possible that during events of O-glycan stress, the expression of these genes, or those involved in the synthesis of the required sugar intermediates, may be down regulated. Interestingly, transcriptional profiling of *C. albicans* demonstrated that when any of the *PMT* genes were deleted, with the exception of *PMT6*, the transcript levels for *DPM1*, encoding the dolichol-phosphate mannose synthase which generates dolichol phosphomannose from GDP-Man and dolichol, increased [9]. At the same time the transcript for *SRB1*, encoding the GDP-mannose pyrophosphorylase, which generates GDP-Man from mannose-1-phosphate and guanosine triphosphate, showed no significant change [9]. Taken together, an increase in *DPM1* expression with no increase in *SRB1 expression*, would reduce the cellular pools of GDP-Man available to the *KTR* family members, with a foreseeable reduction in O-mannose chain-length extension. Interestingly, Cantero et al. also demonstrated that when wild type *C. albicans* was treated with PMTi the transcript levels of numerous genes were regulated differently to when the *PMT* genes were knocked-out [9]. In the presence of inhibitor no changes were observed in *SRB1* transcript levels, while a similar increase in *DPM1* transcript levels was observed, thus, potentially reducing GDP-Man levels by converting it into Dol-P-Man. Other explanations for why we observed a reduction in O-mannose chain-length may be due to reduced expression or activity (due to reduced O-glycosylation) of: the GDP-mannose transporter (Vrg4p), which translocates GDP-mannose from the cytoplasm into the Golgi where the *KTR* members reside, the *KTR* family members themselves, or, some other accessory protein, such as chaperones. Another possible explanation for the apparent occurrence of a higher percentage of shorter chain-length O-linked glycans is that manipulation of the Pmt1p and Pmt2p activities specifically eliminated O-glycan sites which were more accessible to KTR mannosyltransferase activities. As such, by specifically eliminating O-linked glycans with longer chain-lengths, only the shorter, possibly more sterically restricted, glycans remained. A further possibility is that the activities of Pmt4p, Pmt5p or Pmt6p may have been responsible for these shorter glycans and, having eliminated the longer glycans initiated by Pmt1p or Pmt2p, only these shorter glycans remained.

The importance of controlling the O-mannose chain-length on therapeutic proteins has recently been assessed [41]. Cukan and colleagues showed that those proteins expressed in *P* *. pastoris* with mannotriose and longer O-glycans bound to Dendritic Cell-Specific Intercellular adhesion molecule-3-Grabbing Non-integrin (DC-SIGN), whereas proteins with single mannose O-glycans showed DC-SIGN binding that was comparable to that of CHO-produced IgG1, which lacks O-linked mannose [41]. Since DC-SIGN is involved in antigen presentation and the initiation of an immune response, the lack of binding to this receptor is more desirable. Furthermore, recently we have shown that through engineering of O-glycosylation in *P* *. pastoris* , by the introduction of a human-like O-glycosylation pathway, we can enhance the therapeutic application of proteins expressed in *P* *. pastoris* [42].

The methylotrophic yeast, *Pichia* *pastoris* , is an important organism used for the production of therapeutic proteins. Previously our laboratory has described methods for primarily engineering N-glycosylation in this organism. The current study characterizes the *P* *. pastoris* protein-*O*-mannosyltransferase family. In doing so, we have demonstrated that O-glycosylation in *P* *. pastoris* can be manipulated by either knockout of the *PMT* genes or the use of Pmt inhibitor if knockouts are not viable or lead to undesirable phenotypes. As such, these offer a means to reduce non-desirable yeast-like O-glycans. This reduction of O-glycosylation represents an important step forward for the *Pichia* *pastoris* production platform as a suitable system for the production of therapeutic glycoproteins.
